# Validation of Inertial Measurement Units for Measuring Lower-Extremity Kinematics During Squat–Pivot and Stoop–Twist Lifting Tasks

**DOI:** 10.3390/s25185673

**Published:** 2025-09-11

**Authors:** Rutuja A. Kulkarni, Rajit Banerjee, Vicki Z. Wang, Marcel Oliart, Verity Rampulla, Prithvi Das, Alicia M. Koontz

**Affiliations:** 1 Human Engineering Research Laboratories, VA Pittsburgh Healthcare System, Pittsburgh, PA 15206, USA; ruk20@pitt.edu (R.A.K.); rab419@pitt.edu (R.B.); vzw4@pitt.edu (V.Z.W.); mao115@pitt.edu (M.O.);; 2 Department of Bioengineering, Swanson School of Engineering, University of Pittsburgh, Pittsburgh, PA 15261, USA; 3 Department of Physical Medicine and Rehabilitation, University of Pittsburgh Medical Center, Pittsburgh, PA 15206, USA; 4 Department of Rehabilitation Science and Technology, University of Pittsburgh, Pittsburgh, PA 15260, USA

**Keywords:** motion capture, posture, inertial measurement units (IMUs), lifting technique, biomechanics

## Abstract

Optokinetic motion capture (OMC) is the gold standard for measuring the kinematics associated with lifting posture. Unfortunately, limitations exist, including cost, portability, and marker occlusion. The purpose of this study is to evaluate the agreement between OMC and inertial measurement units (IMUs) for quantifying joint kinematics during squat–pivot and stoop–twist lifting tasks. Ten unimpaired adults wearing both IMUs and OMC markers performed 24 lifting trials. Correlation coefficients and Root Mean Square Error (RMSE) between IMU and OMC time-series signals were computed for trunk and lower-extremity joints. Peak values obtained from each system during each trial were analyzed via Bland–Altman plots. Results show high correlations for trunk, knee, and ankle flexion angles (>0.9) and ankle rotation angles (>0.7). Moderate correlation was found for trunk axial rotation and lateral flexion angles (0.5–0.7). RMSE was under 9° for each angle. Biases between systems ranged from 0.3° to 16°. Both systems were able to detect statistically significant differences in peak angles between the two postures (*p* < 0.05). IMUs show promise for recording field data on complex lifting tasks.

## 1. Introduction

Lifting tasks are common during activities of daily living in home and workplace environments and during recreational activities like weightlifting. Such tasks can contribute to musculoskeletal injuries and lower-back pain due to risk factors such as workload, the speed of the lifting maneuver, the vertical and horizontal distance of the weight to the user, repetitions, and improper lifting posture [1,2,3,4,5]. Healthcare professionals, biomechanists, strength and conditioning trainers, and ergonomists have provided prevention strategies to minimize such injuries, especially through proper ergonomics and lifting technique education. For example, in weightlifting, increased knee internal rotation and tibial anterior translation angles during clean and jerk lifts have been shown to predispose weightlifters to an increased risk of anterior cruciate ligament (ACL) injury [6]. Similarly, when comparing squat- and lunge-style jerks, squat-style jerks have been theorized to increase risk of athlete coccyx disease due to the need to cover a greater vertical distance when applying force to a barbell [7]. In workplaces, different lifting posture motions in the sagittal and rotational planes have been proposed. From a sagittal view, the squat posture, compared to the stoop posture, has been recommended as the optimal and safest posture in minimizing lower-back musculoskeletal injuries [8]. The technique for squat lifting involves core engagement and increasing range of motion at the lower limbs, particularly knee and ankle flexion, to optimize a neutral or straight spine that limits trunk flexion [8,9]. Alternatively, stoop posture is associated with increased trunk flexion and decreased range of motion of the lower limbs [8,10]. For rotational motions, pivoting the body using rotational foot translations with the heel of the foot anchored to the ground, rather than twisting from the torso, have been recommended to reduce the risk of musculoskeletal lower-back pain [11]. The evidence for these techniques comes from biomechanical assessments performed with motion analysis using an Optokinetic Motion Capture (OMC) system, which is considered to be the gold standard measurement technique in the field.

One of the largest limitations of OMC analysis is the ability to measure and capture movement outside of a laboratory setting [12]. More recently, wearable OMC systems, such as Inertial Measurement Units (IMUs), have been used to measure movement in more natural, less restricted environments, and have shown promise for real-time musculoskeletal injury detection through validation against standard OMC systems during gait, sports, and various lifting techniques in the workplace [13,14]. For example, during gait, IMU systems have been shown to be a valid tool for assessing knee kinematics compared to OMC systems [15]. In sports involving jump–landing movements, IMU systems provided highly favorable agreement in lower-extremity kinematics in the sagittal plane [16,17]. However, a high degree of error had been reported for kinematics in the frontal plane [17,18]. In the workplace, efforts have been made to validate various lifting techniques in order to mitigate musculoskeletal injury risk by comparing OMC and IMU systems. For example, good agreement was detected for both systems during different lifting methods (snatching, pulling, and pushing) for manhole covering handling when analyzing a user’s back and dominant arm [19]. Similarly, good agreement was also detected during a simple squatting maneuver when analyzing lower-back L5/S1 joint load [20].

Such validation studies often have significant variability in the type of biomechanical outcome measure being assessed (i.e., kinematics, kinetics, and/or EMG) and the joint motion of interest. Thus, although IMU’s have been validated for gait movements, sport-specific maneuvers, and some lifting tasks, they have not been validated for transfer lifting tasks that involve picking up an object and placing it at varying heights and locations. Such tasks involve sagittal and rotational movements and can be a source of musculoskeletal injury, such as lower-back pain, if performed incorrectly from a posture standpoint. Such a comprehensive validation analysis has not been conducted before, and has the potential to provide clinical insight for musculoskeletal lower-back injury prevention and rehabilitation outside of the laboratory setting, addressing a critical gap in evaluating sensor performance for complex musculoskeletal movements. In line with this, the aim of this study was to compare IMUs to gold standard OMC systems for squat–pivot and stoop–twist transfer lifting strategies, analyzing trunk and lower-extremity kinematics across various starting and ending height conditions. Across all height conditions and lifting strategies, we hypothesized that there will be a high degree of system agreement, evidenced by a correlation coefficient of >0.7, and root mean square error (RMSE) less than 10 degrees for trunk, knee, and ankle flexion and rotation angles. These thresholds have been used in other validation studies to assess acceptability [20,21]. We also expected that both systems would be able to identify statistically significant differences in the peak joint angles between the two lifting techniques.

## 2. Materials and Methods

### 2.1. Study Population

Inclusion criteria for participants included being over 18 years of age and having no history of neuro-musculoskeletal disorders or injuries. Participants provided informed consent for this study. Visits were conducted at the Human Engineering Research Laboratories following a study protocol approved by the Human Subjects Subcommittee of the Department of Veterans Affairs.

### 2.2. Procedures

**Figure 1:**
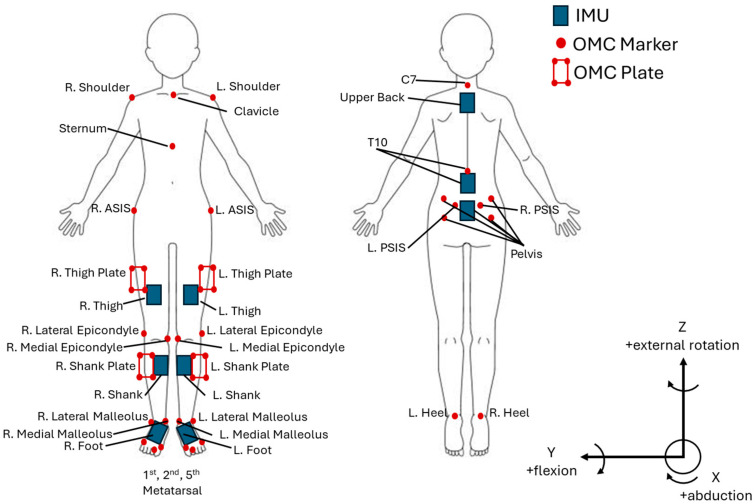
IMU and OMC marker placement.

Participants were outfitted with 28 reflective OMC markers on bony landmarks of the lower extremities, trunk, and neck (Figure 1). Nine Noraxon Ultium Motion IMUs were placed on the feet, legs, and trunk in accordance with manufacturer instructions (Noraxon USA, Scottsdale, AZ, USA) (Figure 1).

For each participant, a walking and standing calibration was captured according to manufacturer instructions and applied to all subsequent IMU trials. Nine Vicon motion capture cameras (Vicon Motion Systems Ltd., Centennial, CO, USA) cameras in a 6.1 m by 6.1 m area were calibrated using a calibration wand prior to data collection. A static trial was captured with the participant standing in the anatomical position. This static trial was applied to all subsequent OMC trials.

**Figure 2:**
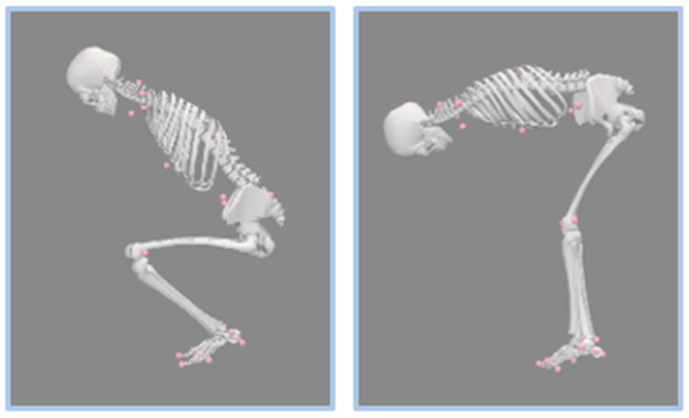
Posture demonstration. The left demonstrates squat posture in the sagittal plane, characterized by high knee flexion and a more upright torso. The right demonstrates stoop posture in the sagittal plane, characterized by low knee flexion and a horizontal torso position. The pink markers represent the OMC system as mentioned above in Figure 1.

Upon completion, participants were given explicit verbal instructions and a demonstration on “incorrect” (stoop–twist) and “correct” (squat–pivot) postures by study personnel. Both positions began from a forward-facing standing posture, with feet placed hip-width apart. Posture education was provided for both sagittal and rotation movements. From a sagittal view, “Stoop” was defined as an emphasis on bending at the hip/lower back and keeping the knees as straight as possible, as opposed to “Squat”, during which participants were directed to bend at the knees and maintain a more upright angle in the trunk (Figure 2). From a rotational view, “pivot” was defined as anchoring the heel of one of the limbs on the ground while rotationally translating the same limb’s mid/forefoot and keeping the contralateral limb anchored. Twisting, conversely, was defined as anchoring both feet at the ground without any foot rotation and twisting at the torso. The squat was paired with the pivot posture, while the stoop was paired with the twisting posture as correct and incorrect posture, respectively. Participants were asked to demonstrate their understanding of the above-mentioned postures, and were given corrections if necessary.

**Figure 3:**
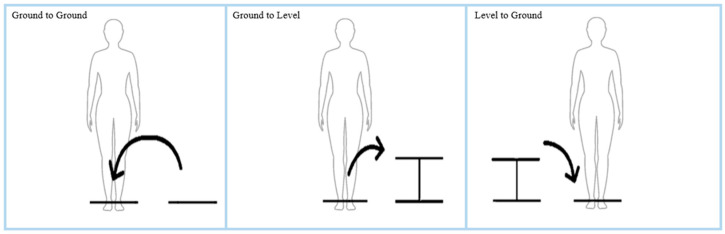
Height condition demonstration from a frontal plane/view. The arrows indicate the direction of the first lift in each trial (with the second lift returning the weight to the starting position).

Participants performed lifting movements using a 4.5 kg box. This load was selected based on prior studies [22,23] demonstrating that comparable weights are sufficient to detect postural and biomechanical differences, while also minimizing the risk of participant injury. These movements were performed under 3 height conditions (Figure 3):

(1) Ground to ground condition: The weight was placed on the floor diagonally in front of the participant, biased toward the left or right. The participant picked up the weight and placed it onto the floor directly in front of them. The weight was lifted again and placed back in the starting position. The process was repeated on the contralateral side (4 trials total, with 2 trials in each direction).
(2) Ground to level condition: In this condition, the weight was lifted from the ground directly in front of the participant and placed at a level height biased to the left or right side (onto a surface that was 52 cm above the ground). The process was repeated on the contralateral side (4 trials total, with 2 trials in each direction).
(3) Level to ground condition: In this condition, weight was lifted from the level height surface (either located from the left or right side) and placed on the ground in front of the participant. The process was repeated on the contralateral side (4 trials total, with 2 trials in each direction).

All of the above-mentioned trials were performed twice (once with squat–pivot and again with stoop–twist) for a total of 24 trials per participant. Trials were performed in a random order. Both IMU and OMC systems simultaneously captured motion during each of the lifting trials. Noraxon IMU data was collected using the Noraxon MyoResearch (MR) software (Version MR 4.2, Noraxon USA, Scottsdale, AZ, USA) at 500 Hz, and OMC data was collected at 100 Hz using Vicon Nexus software (Version 2.9, Vicon Motion Systems Limited, Oxford, UK).

### 2.3. Data Analysis

The IMU data were filtered, and time-series 3D trunk, knee, and ankle joint angles were computed using the Noraxon’s MR 4.2 proprietary software (Noraxon MyoMotion, Noraxon USA Inc.). OMC markers were labeled using the Vicon Nexus 2.9 software. The position data was then imported into Visual 3D (C-Motion, Inc., Germantown, MD, USA) to calculate the 3D trunk, knee, and ankle joint angles. Neutral standing posture in the anatomical position was defined as zero degrees for all measured joint angles (e.g., trunk flexion, rotation, and bending all at zero degrees, zero degrees with full knee extension, and zero degrees of ankle flexion and rotation). Trunk flexion is defined as positive from the vertical. Trunk lateral flexion and axial rotation are defined as positive moving towards the right of midline and negative moving towards the left of midline. Ankle dorsiflexion is defined as positive, while planar flexion is defined as negative. Ankle rotation refers to ankle abduction and adduction, where abduction is defined as positive. All knee and ankle angles were calculated using the International Society of Biomechanics (ISB) standards [24].

A custom MATLAB code (Version R2023b, MathWorks Inc., Natick, MA, USA) was written to perform data analysis on the calculated angles. Data from Visual3D was filtered in MATLAB using a fourth order Butterworth filter with a cutoff frequency of 10 Hz [25]. IMU angles were down-sampled to 100 Hz to match the sampling rate of the OMC angles. Cross-correlation (XCORR) was used to determine the optimal lag between the two signals [17]. Signals were synchronized by shifting IMU time-series joint angle data to start and end at the same time as the OMC signal. Pearson’s correlation coefficients between the OMC and IMU signals and RMSE were calculated for each joint angle (trunk forward flexion, trunk lateral flexion, trunk axial rotation, left and right knee flexion, ankle flexion, and ankle rotation) of each lifting trial for each participant. Evaluation of calculated Pearson’s correlation coefficients are as follows [26]: very high (0.90–1.00), high (0.70–0.90), moderate (0.50–0.70), low (0.30–0.50), and negligible (0.00–0.30). Correlation coefficients and RMSE values were averaged across all of the lifting trials. One maximum (peak) value was extracted from each OMC and IMU trial for all observed joints. For trunk lateral flexion and trunk axial rotation, an absolute value of the maximum and minimum peaks was determined, and the larger magnitude value of the two was used as the peak value.

### 2.4. Statistical Analysis

Peak values for each joint angle and posture were averaged across trials for each subject. A paired *t*-test was conducted to compare average peak joint values between the squat–pivot and stoop–twist postures for each system (n = 10). A Bland–Altman [27] analysis was conducted comparing peak values of each joint angle for each lift trial across both OMC and IMU systems. Prior to calculating a 95% confidence interval and creating Bland–Altman plots, outliers in each joint angle were removed if the difference between the two systems was farther than three standard deviations from the mean difference, indicating potential technical errors caused by marker drop out or occlusion or IMU sensor slippage. Bland–Altman (BA) plots were created using the Bland–Altman MATLAB package [28]. Joint angle peaks were analyzed using linear regression within the same MATLAB framework. The model presented values, including a line of best fit and a coefficient of determination ($r^{2}).$ The interpretation of the square root of the $r^{2}$ values follows similar thresholds to the Pearson’s correlation coefficient.

## 3. Results

Ten adults (five females and five males) participated in this study. This number was selected based on prior studies [22,29] that were appropriately able to detect differences between systems. Participants had a mean (±standard deviation) age of 23 ± 3 years old, height of 172.5 ± 9.7 cm, and weight of 73.3 ± 10.0 kg. The results are presented by joint angle for ease of display and interpretation.

### 3.1. Trunk Forward Flexion

The time-series curves for trunk forward flexion showed very high agreement between the two systems for both postures. Furthermore, trunk forward flexion yielded the highest correlation value between both IMU and OMC systems’ time-series signals for all of the assessed joint angles (0.98). The RMSE values for the time-series curves were less than 9° (Table 1).

**Figure 4:**
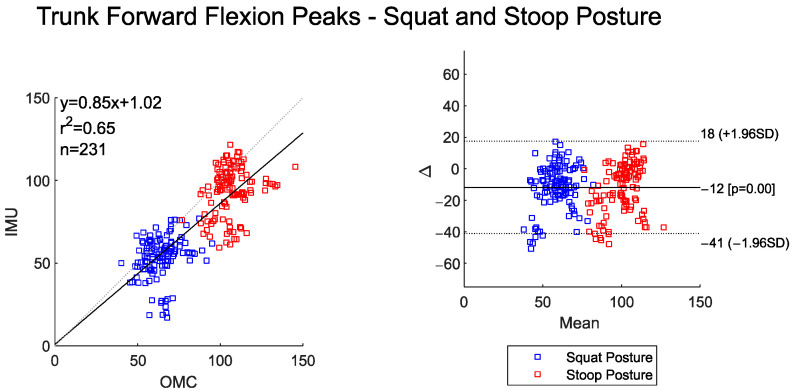
Scatter plot of the IMU and OMC peak angles with line of best fit (**left**), and Bland–Altman plot (**right**) of the peak trunk flexion angles for both squat and stoop postures. No outliers were removed in this dataset.

Both systems detected significantly lower peak trunk flexion angles for the squat–pivot versus stoop–twist posture (Table 2). The peak angle commonly occurred before the object was lifted from the ground or immediately after the object was released on the ground, depending on the lifting conditions of the observed trial (ground to ground or level to ground). This can further be observed in the BA plot in Figure 4, which shows a cluster of data into two distinct groups (lower peaks for squat postures and higher peaks for stoop postures). However, peak trunk flexion angles for IMU are approximately 12° lower than OMC systems. The relatively equal distribution of points around the mean referenced line in the BA plot (y = −12) indicates no proportional bias between the two systems. The $r^{2}$ of 0.65 indicates high agreement between the systems for peak trunk flexion angles.

### 3.2. Trunk Lateral Flexion and Axial Rotation

The time-series curves for trunk lateral flexion between the OMC and IMU systems show moderate agreement across both postures. The RMSE values for trunk lateral flexion are around 8° (Table 3). Both the IMU and OMC systems detected statistically lower peak (maximum) trunk lateral flexion angles for the squat–pivot compared to the stoop–twist posture (Table 4).

**Figure 5:**
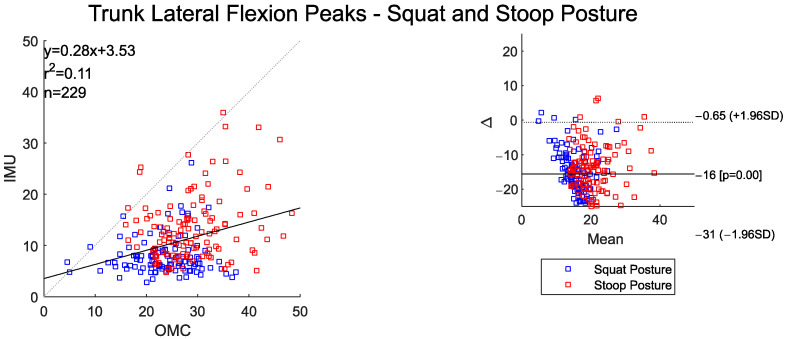
Scatter plot of the IMU and OMC peak angles with line of best fit (**left**), and Bland–Altman plot (**right**) of peak trunk lateral flexion angles for both squat and stoop postures. Two outliers were removed.

Mean peak trunk lateral flexion angles were lower for IMU (~16°) compared to OMC (Figure 5) systems. There was low correlation between the two systems for these peak angles. The scatter plot and BA plot indicate a tendency for the IMU system to underestimate the peak trunk lateral flexion angles at higher OMC peak angles.

**Figure 6:**
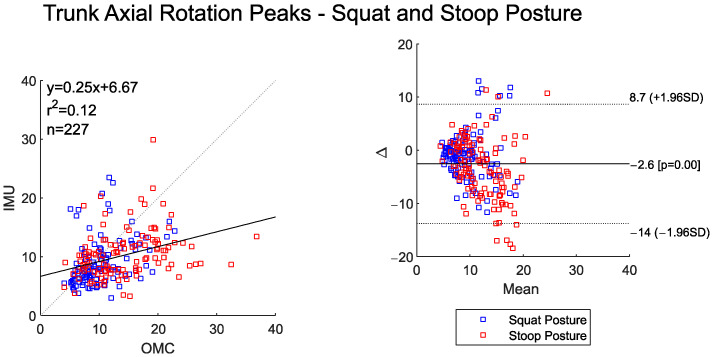
Scatter plot of the IMU and OMC peak angles with line of best fit (**left**), and Bland–Altman plot (**right**) of peak trunk axial rotation angles for both squat and stoop postures. Four outliers were removed.

There was weak agreement in the trunk axial rotation time-series curves between the systems (Table 3). The IMU system detected significantly higher trunk rotation angles during the stoop–twist posture compared to squat–pivot posture (*p* = 0.040), while the OMC system did not (*p* = 0.668) (Table 4). There was also low agreement in the peak trunk axial rotation angles between the systems, and the scatter plot shows the IMU system underestimating the OMC more-so at higher joint angle values (Figure 6). The BA plot of the peak trunk rotation angles shows an overall small bias between the two systems (~2.6°), but increasing variability in the mean differences as the peak angles increase.

### 3.3. Knee Flexion

The time-series curves for left and right knee flexion show very high agreement between the two systems across both postures (Table 5). RMSE values were between 4° and 8°. Both systems detected significantly higher peak left and right knee flexion for the squat–pivot compared to stoop–twist postures (Table 6).

**Figure 7:**
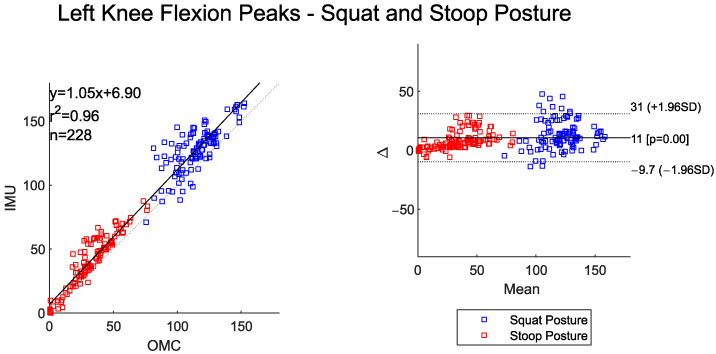
Scatter plot of the IMU and OMC peak angles with line of best fit (**left**), and Bland–Altman plot (**right**) of peak left knee flexion angles for both squat and stoop postures. Three outliers were removed.

**Figure 8:**
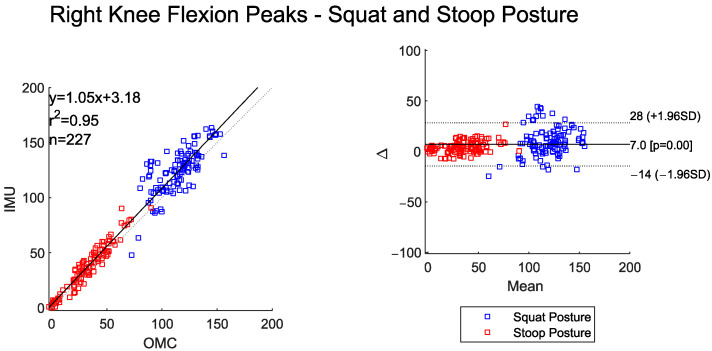
Scatter plot of the IMU and OMC peak angles with line of best fit (**left**), and Bland–Altman plot (**right**) of peak right knee flexion angles for both squat and stoop postures. Four outliers were removed.

These posture-based differences are clearly visible in Figure 7 and Figure 8, where both the scatter and BA plots show two distinct groups of data. Peak knee flexion angles were almost perfectly correlated between the two systems. The IMU system, however, consistently overestimated peak values relative to OMC by about 11 degrees for left knee flexion and 7 degrees for right knee flexion. The BA plot shows evidence of proportional bias, with larger deviation between systems occurring at larger magnitude peak left and right knee flexion angles.

### 3.4. Ankle Flexion

The time-series data between IMU and OMC systems for both right and left ankle flexion showed high-to-very-high agreement (Table 7). Overall RMSE values were under 3°. Both systems detected statistically higher peak left and right ankle flexion for the squat–pivot compared to the stoop–twist posture (Table 8).

**Figure 9:**
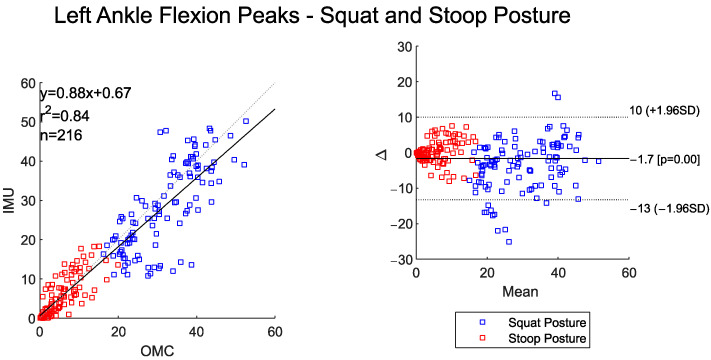
Scatter plot of the IMU and OMC peak angles with line of best fit (**left**), and Bland–Altman Plot (**right**) of peak left ankle flexion angles for both squat and stoop postures. Four outliers were removed.

**Figure 10:**
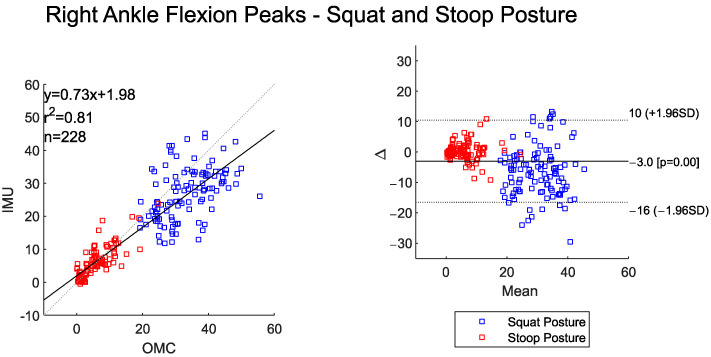
Scatter plot of the IMU and OMC peak angles with line of best fit (**left**), and Bland–Altman Plot (**right**) of peak right ankle flexion angles for both squat and stoop postures. Three outliers were removed.

The scatter and BA plots for left and right ankle flexion also illustrate two distinctly separate groups, one for each assessed posture (Figure 9 and Figure 10). The IMU system overall measured slightly lower peak ankle flexion values for the left and right side when compared to the OMC (BA plot bias for left ankle −1.7° and right ankle −3°). The BA plots show proportional bias, with greater system differences at higher peak angles. The left and right peak ankle flexion angles shows a high correlation between the two systems (Figure 9 and Figure 10).

### 3.5. Ankle Rotation

The time-series left and right ankle rotation angle data shows moderate agreement between the two systems (Table 9). The RMSEs were also low (<3.5°) for ankle rotation angles computed between the IMU and OMC systems (Table 9). Both systems detected significantly higher right and left peak ankle rotation angles during the squat–pivot posture compared to the stoop–twist posture (Table 10).

**Figure 11:**
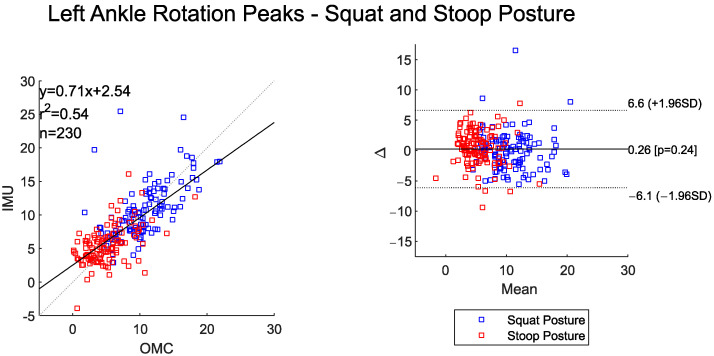
Scatter plot of the IMU and OMC peak angles with line of best fit (**left**), and Bland–Altman plot (**right**) of peak left ankle rotation angles for both squat and stoop postures. One outlier was removed.

**Figure 12:**
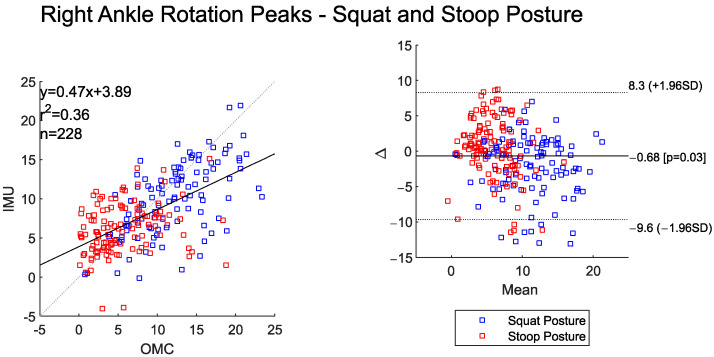
Scatter plot of the IMU and OMC peak angles with line of best fit (**left**), and Bland–Altman plot (**right**) of peak right ankle rotation angle for both squat and stoop postures. Three outliers were removed.

The BA plot shows a small overall system bias for the left- and right-side peak ankle rotation angles (<1°). The scatter plots show moderate-to-high agreement between the peak values (Figure 11 and Figure 12). The IMU system had a tendency to underestimate ankle rotation relative to the OMC at higher joint angle values and overestimate the OMC system at lower ankle rotation angles.

## 4. Discussion

### 4.1. Time-Series Joint Angles: Correlations and RMSE

This study aimed to validate an IMU system against an OMC system for motion capture during squat–pivot and stoop–twist lifting tasks by evaluating the trunk and lower-extremity joint kinematics agreement between the two systems. The highest agreement was found for motions in the sagittal plane. Since previous validation studies have primarily focused on gait applications, direct comparisons of our results with prior work is difficult. However, high sagittal plane agreement is consistent with previous validation studies that assessed IMU and OMC systems for non-lifting tasks [17,30,31]. Lower correlations were found in the frontal and transverse planes, with the lowest correlation being found during trunk rotation. These differences may be due in part to the IMU calibration protocol within the Noraxon software, which involves calibration over the subject’s walking gait. Currently, this is the most extensive calibration offered by the software. Therefore, it is most accurately calibrated for the subsequently smaller ranges of motion in the frontal/transverse planes, and not the greater angles seen in the lifting motions measured here.

Previous studies have noted acceptable RMSE differences to be up to 10° when examining gait and other activities [18,19,32]. All of the angle RMSE values found in this study were under 9°. Knee flexion angles for some postures and all ankle angles, however, showed an RMSE of under 5°. Overall, the moderate-to-high agreement and low RMSE found in the time-series data across the angles provides good justification for using IMUs in lieu of OMC systems for studies involving squat and stoop postures and the types of lifting tasks performed in this study.

### 4.2. Peak Joint Angles: Correlations and Biases

Results for the peak joint angles follow similar patterns mentioned above, with sagittal plane motions, particularly trunk, knee, and ankle flexion, showing stronger agreement than transverse/frontal plane motions. Researchers in this study chose to focus on the peak values for the paired t-test, linear regression, and BA analysis, as peak joint kinematic values are oftentimes used to measure risk of injury, whether it be solely through joint angles or by performing inverse dynamics calculations to assess joint moments and forces. Biases detected in the BA analysis ranged in magnitude between 0.26 and 16°. This is consistent with a previous study that aimed to validate two motion-capture systems for sports-related tasks, with their study noting a range in bias between 0 and 16° [33]. The IMUs tended to underestimate the trunk angles and overestimate the knee angles. Ankle angles were more similar between the two systems with regard to overall bias. For several of the angles, the scatter and BA plots indicate a proportional bias. IMUs tended to underestimate the angles at higher OMC angles. As a result, researchers should remain cautious when using IMUs and interpreting peak values in movements involving larger ranges of motion, such as deep squatting.

The differences in calculated joint angles between systems can be attributed to several factors. For one, the effect of soft-tissue artifacts likely produces different errors between the OMC and IMU systems due to the differences in how the sensors/optical markers are placed on the body: while OMC optical markers are on bony landmarks on every body segment (e.g., the malleolus, epicondyle, ASIS, etc.), the thigh and lower thoracic IMU sensors are placed over areas of thicker soft tissue. This could amplify soft tissue artifacts on the kinematics calculated from these sensors, especially at the ends of joint ranges of motion (fully flexed knee or trunk), where soft tissue is most displaced, as compared to during calibration.

Other differences in how each system performs kinematic analysis could additionally lead to discrepancies between the systems. IMU data calculations rely on a functional walking calibration model, while the OMC system relies on a static calibration. A different IMU calibration process involving motions more relevant to the study goals may help rectify some of the issues found with the axial and lateral rotations. For the specific IMU system utilized for this study, a walking calibration was the most thorough calibration mode available. The IMU system relies on orientations directly from local coordinate systems in the sensors to compute the angles, while the OMC system builds a local coordinate system from body markers (position data) in reference to a global coordinate system. Furthermore, it is possible that the two software systems used to compute the kinematics (Noraxon MR and Visual 3D) make different model assumptions [30]. Differences exist in the filtering used for both systems, with the IMU system reportedly using a Kalman filter, while a Butterworth filter was used for the OMC data. Additionally, IMUs calculate joint angle via mathematical integration of the angular velocity values directly measured by the sensors. The process of integration amplifies any errors in the measurement, and the error-to-signal ratio is greatest when the velocity is smaller—i.e., at the peaks of joint angle. Therefore, the integrative process used to calculate kinematics from IMU data may produce a greater error at peaks and valleys in joint angles.

Others have tried to mitigate differences by performing repeated calibrations of the OMC and IMU systems (static calibration) between trials [32]; however, the results show that a single calibration file can be used without meaningfully impacting the results. Other work has also attempted to allow for a more ‘apples-to-apples’ comparison by calibrating IMU system data to concurrently measured OMC data; this would then allow for the adjustment of all following IMU data to ostensibly better approach the values that may have been measured by a validated OMC [34].

### 4.3. Posture Differences Between Systems

Both systems were able to detect statistically significant differences in peak values between squat–pivot and stoop–twist for each joint angle. Squat–pivot yielded a more neutral trunk alignment (less trunk flexion, axial rotation, and lateral bending) coupled with greater knee and ankle flexion and more ankle rotation due to pivoting of the feet, as expected. The only exception was for the OMC system for trunk axial rotation, which did not result in a statistically significant difference; however, mean differences for both systems overall were small (<2°), but were in the expected direction. The relatively lower variances around the means made it easier to detect statistical differences with the IMU system. This provided promising results for the IMU system in terms of it being able to identify expected differences between postures in future applications.

### 4.4. Implications

The results from our study support the potential applicability of IMU systems for measuring joint kinematics in real-world settings. For example, healthcare personnel or caregivers performing similar types of maneuvers that were tested in this study are prone to developing lower-back musculoskeletal injuries [35]. From a postural standpoint, IMU’s have the potential to accurately measure lower-back joint kinematics in the sagittal plane, and can thus likely differentiate riskier stoop postures vs. squat postures. However, given our above results, the IMU systems may be less likely to yield accurate results for pivoting or twisting motions, particularly at higher ranges of motion. Thus, it depends on the desired motions being captured, study objectives, and tolerance for error as to whether IMUs can be used in a protocol in lieu of the OMC system. Future researchers can utilize the findings presented here to help inform the decision as to whether using IMUs is appropriate for their efforts. Given the biases found between the systems, particularly when it comes to higher-magnitude joint angles, it is not recommended to make direct comparisons between the absolute kinematic values obtained via OMC versus IMU systems. However, our study shows that IMUs work well for detecting differences in motion strategies in repeated-measures study designs.

Apart from injury prevention assessment, IMU systems can also have applicability for rehabilitation purposes by allowing for clinicians and physical/occupational therapists to more easily (in comparison to an OMC lab or setup) assess patient biomechanics, which can guide or evaluate the effectiveness of clinical interventions. Using IMU systems also makes it possible to conduct assessments in real-world settings like ay home, work, or other community-based environments, potentially enabling more clinically meaningful data to be collected.

### 4.5. Limitations

There are several limitations worth noting within our study. This study included ten young healthy adults with low body mass index (BMI). Thus, this is not directly characterizable to older populations who experience different biomechanical and physiological demands. From a BMI standpoint, both sensor and OMC performance may be altered and susceptible to artifact in individuals with increased BMI due to the presence of soft tissue [36]. It is difficult to ascertain, however, to what degree the effects have relative to one another from a validation standpoint. Nonetheless, the artifact effects from both systems can affect real-world applicability.

Some outliers were observed for each joint angle. These outliers can likely be attributed to noise or a data collection error (OMC marker occlusion, IMU sensor slippage, or IMU hardware/software issues) in one or both systems. The percentage of trials that were removed was very low (<1.7% of trials per joint), indicating the robustness of the instrumentation for measuring multi-planar motions. Ankle and knee flexion time-series correlation values were similar to the values calculated in previous non-lifting validation studies [17,30,31].

Researchers noted difficulties using the OMC system during data collection, as some ankle markers repeatedly fell off during squat–pivot transfers, likely due to the significant movement/deformation of the soft tissue that the markers adhere to, or the marker falling off due to contact with the weight being lifted. This caused researchers to have to recalibrate the OMC system repeatedly for some participants. The same issue did not arise for the IMU system, as the IMUs use a strap system on the lower extremities (Figure 1), and the IMUS that are used to calculate ankle angles (shank, foot) are located on bony areas. This does not affect the results of the study, but, rather, it is a notable limitation in using an OMC system. This study also had a small sample size, and therefore having a larger group can help increase the overall reliability and applicability of the results to the general population. Future studies should address these components while expanding kinematic analysis to include kinetic data validation (i.e., force platform, pressure insoles, electromyography), which were not included in the present study. This could ultimately provide a more comprehensive biomechanical analysis that could potentially be more clinically relevant.

## 5. Conclusions

IMUs show promise for detecting complex multi-planar motions, but are dependent on the plane of motion, showing higher validity for sagittal plane motions compared to transverse and frontal plane motions. Further, IMUs can detect differences between squat–pivot posture and stoop–twist posture. IMUs offer a useful alternative to OMC due to practicality, low cost, and feasibility for use outside of the laboratory setting. Outside laboratory use can allow healthcare professionals to measure long-term and day-to-day use during real-world biomechanical applications, and thus can provide clinical insights for injury prevention and rehabilitation—data that would otherwise be difficult to capture with in-lab assessments.

## Figures and Tables

**Table 1:** Mean and standard deviation (SD) of the Pearson correlation coefficients and RMSE for the time-series trunk flexion angles.

Joint Angle	Posture	Overall Correlation Coefficient (SD)	RMSE (SD) (°)
Trunk Forward Flexion	Squat–pivot	0.97 (0.05)	8.06 (5.67)
	Stoop–twist	0.99 (0.01)	8.72 (4.64)
	Overall	0.98 (0.04)	8.33 (5.25)

**Table 2:** Mean and standard deviation (SD) of the peak trunk flexion angles assessed for each system and for each posture.

Joint Angle	System	Squat–Pivot Mean (SD) Value (°)	Stoop–Twist Mean (SD) Value (°)	Two-Sided *p*-Value (n = 10)
Trunk Forward Flexion	IMU	53.20 (12.36)	94.31 (13.76)	<0.001
	OMC	64.30 (6.87)	106.43 (9.06)	<0.001

**Table 3:** Mean and standard deviation (SD) of the Pearson correlation coefficients and RMSE for the time-series trunk lateral flexion and trunk axial rotation angles.

Joint Angle	Posture	Overall Correlation Coefficient (SD)	RMSE (SD) (°)
Trunk Lateral Flexion	Squat–pivot	0.53 (0.28)	8.19 (5.87)
	Stoop–twist	0.73 (0.22)	7.37 (2.65)
	Overall	0.63 (0.27)	7.85 (4.80)
Trunk Axial Rotation	Squat–pivot	0.53 (0.28)	5.60 (7.25)
	Stoop–twist	0.46 (0.25)	5.58 (6.57)
	Overall	0.50 (0.27)	5.59 (5.82)

**Table 4:** Mean and standard deviation (SD) of the peak trunk lateral flexion and trunk axial rotation angles for each system and for each posture.

Joint Angle	System	Squat–Pivot Mean (SD) Value (°)	Stoop–Twist Mean (SD) Value (°)	Two-Sided *p*-Value (n = 10)
Trunk Lateral Flexion	IMU	8.10 (1.69)	13.56 (3.89)	0.002
	OMC	23.85 (3.91)	29.11 (4.26)	<0.001
Trunk Axial Rotation	IMU	9.02 (3.10)	10.48 (2.51)	0.040
	OMC	14.43 (6.98)	15.88 (7.55)	0.668

**Table 5:** Mean and standard deviation (SD) of the Pearson correlation coefficients and RMSE for the time-series left and right knee flexion angles.

Joint Angle	Posture	Overall Correlation Coefficient (SD)	RMSE (SD) (°)
Left Knee Flexion	Squat–pivot	0.98 (0.09)	7.90 (3.60)
	Stoop–twist	0.93 (0.13)	4.25 (2.82)
	Overall	0.95 (0.12)	2.74 (1.81)
Right Knee Flexion	Squat–pivot	0.98 (0.05)	6.74 (2.76)
	Stoop–twist	0.91 (0.19)	4.34 (2.63)
	Overall	0.94 (0.15)	4.76 (2.78)

**Table 6:** Mean and standard deviation (SD) of the peak left and right knee flexion angles assessed for each system and for each posture.

Joint Angle	System	Squat–Pivot Mean (SD) Value (°)	Stoop–Twist Mean (SD) Value (°)	Two-Sided *p*-Value (n = 10)
Left Knee Flexion	IMU	129.61 (17.22)	39.11 (19.18)	<0.001
	OMC	115.39(15.20	30.95 (14.85)	<0.001
Right Knee Flexion	IMU	125.50 (16.93)	35.85 (18.11)	<0.001
	OMC	113.56 (13.03)	31.62 (16.73)	<0.001

**Table 7:** Mean and standard deviation (SD) of the Pearson correlation coefficients and RMSE for the time-series left and right ankle flexion angles.

Joint Angle	Posture	Overall Correlation Coefficient (SD)	RMSE (SD) (°)
Left Ankle Flexion	Squat–pivot	0.97 (0.06)	3.46 (2.00)
	Stoop–twist	0.93 (0.09)	2.22 (1.55)
	Overall	0.95 (0.09)	2.74 (1.81)
Right Ankle Flexion	Squat–pivot	0.85 (0.17)	3.62 (2.02)
	Stoop–twist	0.88 (0.17)	1.94 (1.68)
	Overall	0.89 (0.17)	2.29 (1.87)

**Table 8:** Mean and standard deviation (SD) of the peak left and right ankle flexion angles for each system and for each posture.

Joint Angle	System	Squat–Pivot Mean (SD) Value (°)	Stoop–Twist Mean (SD) Value (°)	Two-Sided *p*-Value (n = 10)
Left Ankle Flexion	IMU	28.03 (10.13)	5.93 (3.59)	<0.001
	OMC	33.11 (8.99)	6.21 (3.73)	<0.001
Right Ankle Flexion	IMU	27.30 (7.66)	5.36 (2.77)	<0.001
	OMC	35.10 (7.31)	5.25 (3.28)	<0.001

**Table 9:** Mean and standard deviation (SD) of the Pearson correlation coefficients and RMSE for the time-series left and right ankle rotation angles.

Joint Angle	Posture	Overall Correlation Coefficient (SD)	RMSE (SD) (°)
Left Ankle Rotation	Squat–pivot	0.78 (0.19)	3.35 (1.38)
	Stoop–twist	0.76 (0.20)	2.57 (1.64)
	Overall	0.77 (0.20)	2.74 (1.81)
Right Ankle Rotation	Squat–pivot	0.75 (0.20)	3.46 (1.77)
	Stoop–twist	0.75 (0.22)	3.19 (1.96)
	Overall	0.75 (0.21)	3.24 (1.91)

**Table 10:** Mean and standard deviation (SD) of the peak left and right ankle rotation angles assessed for each system and for each posture.

Joint Angle	System	Squat–Pivot Mean (SD) Value (°)	Stoop–Twist Mean (SD) Value (°)	Two-Sided *p*-Value (n = 10)
Left Ankle Rotation	IMU	10.81 (1.69)	5.46 (1.04)	<0.001
	OMC	10.69 (2.03)	4.99 (1.78)	<0.001
Right Ankle Rotation	IMU	10.21 (2.93)	6.17 (1.83)	0.002
	OMC	12.66 (3.16)	5.60 (2.47)	<0.001

## Data Availability

De-identified data can be made available upon request from the corresponding author (AK) and with an approved Data Use Agreement through the VA.

## Abbreviations

The following abbreviations are used in this manuscript:

IMU	Inertial measurement unit
OMC	Optokinetic motion capture
XCORR	Cross correlation
RMSE	Root mean square error
r^2^	Coefficient of determination
